# Characterization of complete chloroplast genome of *Salvia honania* L. H. Bailey (Lamiaceae)

**DOI:** 10.1080/23802359.2022.2108347

**Published:** 2022-08-10

**Authors:** Zi-Xuan Wang, Yan-Bo Huang, Fu-Hai Yuan, Jun-Jie Lin, Han-Wen Xiao, Dong-Feng Yang, Yu-Kun Wei

**Affiliations:** a College of Life Sciences and Medicine, Key Laboratory of Plant Secondary Metabolism and Regulation in Zhejiang Province, Zhejiang Sci-Tech University, Hangzhou, China; b Eastern China Conservation Centre for Wild Endangered Plant Resources, Shanghai Chenshan Botanical Garden, Shanghai, China; c Changzhou Menghe Shuangfeng Chinese Herbal Medicine Technology Co. LTD, Changzhou, China; d Zhejiang Sci-tech University Shaoxing Biomedical Research Institute Co. LTD, Shaoxing, China; e Shanghai Botanical Garden, Shanghai, China

**Keywords:** *Salvia honania* L. H. Bailey, chloroplast genome, phylogeny

## Abstract

*Salvia honania* L. H. Bailey is an endemic species, mainly distributed in Henan and Hubei provinces in China. The first complete chloroplast genome of *Salvia honania* was sequenced and assembled in this study. The genome is 151,559 bp in length and contains 132 encoded genes in total, including 87 protein-coding genes, eight ribosomal RNA genes, and 37 transfer RNA genes. The phylogenomic analysis showed that *Salvia honania* was closely related to *Salvia meiliensis* according the current sampling extent.

*Salvia honania* L. H. Bailey 1920 (Lamiaceae) is a perennial herb of the genus *Salvia*, distributed in Hubei and Henan in China (Cai et al. 1998). The genus *Salvia* is the largest genus in the family LamiaceaeLabiatae, and there are three diverse distribution centers in the world, with China is being the center of diversity in East Asia (Walker and Sytsma 2007), especially in Yunnan, Sichuan province and the Hengduan Mountains, where the species diversity and endemism ratio are the highest. *Salvia honania* blooms in May and its flowers are coppery, with stamens and pistil exserted from the corolla about 1 cm. Previous studies have found that it can promote blood circulation, regulate menstruation, remove blood stasis and relieve pain (Cai et al. 2009; Yan et al. 2012). The extracts of *Salvia* leaves have definite antioxidant effects and radical scavenging activities (Qin et al. 2006). It is reported that the sage may have a therapeutic effect on Alzheimer’s disease (Ovidi and Masci 2018).

However, the complete chloroplast (cp) genome sequence of *Salvia honania* has not been reported so far. The chloroplast genome is mostly maternally inherited in angiosperms and is largely free of recombination, while it retains many single-copy genes inherited during evolutionary history, which is advantageous for determining species relatedness by studying the chloroplast genome (Wolfe et al. 1987; Clegg et al. 1994). And the plastid genome will contribute to develop protection strategy for this species. In this study, we reported the complete cp genome sequence of *Salvia honania*.

The leaves of *Salvia honania* was collected from Xinyang, Henan, China (GPS: E114°04′03.15″, N31°49′46.46″). The specimen was deposited at Herbarium of Shanghai Chenshan Botanical Garden (CSH) (Yukun Wei, ykwei76@hotmail.com) under the voucher number S0356. The total genomic DNA was extracted from its silica dried leaves using DNA Plantzol Reagent (Invitrogen, Carlsbad, CA, USA) in accordance with the manufacturer’s instructions. The plastome sequences were generated using the Illumina HiSeq 2500 platform (Illumina Inc., San Diego, CA, USA). In total, ca. 11.6 million high-quality clean reads (150 bp PE read length) were generated with adaptors trimmed. Aligning, assembly, and annotation were conducted using GetOrganelle v1.7.0c, BLAST, GeSeq (Michael et al. 2017) and GENEIOUS v11.0.5 (Biomatters Ltd, Auckland, New Zealand).

The full length of *Salvia honania* chloroplast sequence (GenBank Accession No.NC_058852) is 151,559 bp, consisting of a large single copy region (LSC with 82,817 bp), a small single copy region (SSC with 17,576 bp), and two inverted repeat regions (IR with 25,583 bp). The GC content of *S.honania* chloroplast genome was 43.1%. A total of 132 genes were annotated in the genome (87 protein-coding genes, eight rRNA genes, and 37 tRNA genes). Seventeen genes had two copies, which were comprised of seven protein-coding genes (*ndhB, rps7, rpl2, ycf2, ycf15, rpl23, rps12*), six tRNA genes (*trnV-GAC, trnI-GAU, trnA-UGC, trnR-ACG, trnN-GUU, trnL-CAA*), and all four rRNA species (*rrn16, rrn23, rrn4.5, rrn5*). In the genome, eight protein-coding genes (*atpF, rpl2, ndhB, ndhA, rps16, rps12, rpoC1, petD*) had one intron, and *ycf3* gene contained two introns.

A total of 29 microsatellites (SSRs) were identified in the *Salvia honania* cp genome using MISA. Among them, 26 of these were located in the LSC regions and three in the SSC regions. The majority of the SSRs (25/29) were A or T repeats, which was consistent with the A/T-richness in the complete cp genome. The complete chloroplast genome sequence provides the necessary data for the study of organ development genes and phylogenetic studies of the Lamiaceae family. The results of this study could be useful for further studies on *Salvia honania*.

To confirm the phylogenetic position of *Salvia honania,* we obtained fourteen published chloroplast genomes of Lamiaceae and one accession of Verbenaceae from NCBI (https://www.ncbi.nlm.nih.gov). The sequence alignment was conducted using MAFFT v7.3 (Katoh and Standley 2013). The maximum likelihood (ML) phylogenetic analyses were constructed using IQTREE v1.6.7 (Nguyen et al. 2015), with the best selected TVM + F+R2 model and 5000 bootstrap replicates. The phylogenetic tree revealed that *Salvia honania* was closely related to *Salvia meiliensis* according to the current sampling extent (Figure 1).

**Figure 1.**
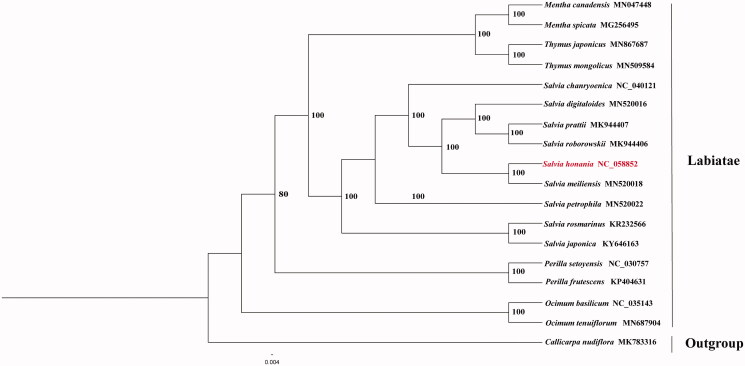
Phylogeny of Labiatae based on complete chloroplast genomes (accession numbers were listed behind each taxon. Statistical support values were showed on nodes).

## Data Availability

The genome sequence data that support the findings of this study are openly available in GenBank of NCBI (https://www.ncbi.nlm.nih.gov) under the accession no. NC_058852. The associated BioProject, SRA, and Bio-Sample numbers are PRJNA761213, SRR15734230, and SAMN21246184, respectively.
